# Different models of pharmaceutical services and care in primary healthcare clinics in the Eastern Cape, South Africa: Challenges and opportunities for pharmacy practice

**DOI:** 10.4102/phcfm.v12i1.2323

**Published:** 2020-07-27

**Authors:** Amy C. Bobbins, Susan Burton, Teri-Lynne Fogarty

**Affiliations:** 1 Department of Pharmacy, Faculty of Health Sciences, Nelson Mandela University, Port Elizabeth, South Africa; 2 Pharmacy Practice Division, Faculty of Pharmacy, Rhodes University, Grahamstown, South Africa

**Keywords:** primary health care re-engineering, pharmaceutical services, pharmaceutical care, dispensing models, task-shifting

## Abstract

**Background:**

Primary health care (PHC) re-engineering forms a crucial part of South Africa’s National Health Insurance (NHI), with pharmaceutical services and care being crucial to treatment outcomes. However, owing to a shortage of pharmacists within PHC clinics, task-shifting of the dispensing process to pharmacist’s assistants and nurses is common practice. The implications of this task-shifting process on the provision of pharmaceutical services and care remains largely unstudied.

**Aim:**

The study aimed to explore the pharmacist-based, pharmacist’s assistant-based and nurse-based dispensing models within the PHC setting.

**Setting:**

The Nelson Mandela Bay Health District, South Africa.

**Methods:**

A mixed methods approach was utilised comprising of Phase 1: a pharmaceutical services audit to analyse pharmaceutical service provision and Phase 2: semi-structured interviews to describe the pharmaceutical care provision within each dispensing model thematically.

**Results:**

Pharmaceutical services partially fulfilled minimum standards within all models, however, challenges exist that limit the quality of these services. Phase 2 showed that the provision of pharmaceutical care within all models was restricted by context-related constraints, thus patient-centred activities to underpin pharmaceutical services were limited.

**Conclusion:**

Although pharmaceutical services may have been available for all models, compromised quality of these services impacted overall quality of care. Limited pharmaceutical care provision was evident within each dispensing model. The results raised concerns about the current utilisation of pharmacy personnel, including the pharmacist, within the PHC setting. Further opportunities exist, if constraints allow, for the pharmacist to contribute to better patient-centred care.

## Introduction

Since 1994, South Africa has made progress towards a more equitable and united healthcare system for all South Africans, based on ideals of Universal Healthcare Coverage (UHC). These attempts have culminated in the implementation of the National Health Insurance (NHI), with primary health care (PHC) re-engineering being a major emphasis in the preparatory first phase of implementation. Considering South Africa’s quadruple burden of disease (communicable diseases, non-communicable diseases, injuries and HIV), this reform justly aims to emphasise the strengthening of preventative and promotive PHC in conjunction with curative services.^1,2^ Strengthening PHC, and its core principles of equity, affordability, effectiveness and efficiency, has been identified as an effective way of strengthening health systems in low- and middle-income countries (LMICs).^2^ Strengthening health systems aligned to PHC core principles promotes patient-centred health systems, providing care closer to patients aligned with social determinants of health.^3,4^

Thus, PHC strengthening initiatives have become of crucial importance to the health systems in many African countries and beyond. In South Africa, the revitalisation of PHC through the implementation of Operation Phakisa and the development of the Ideal Clinic (IC) concept, saw the growth in capacity and quality of health facilities with outreach and community-based services.^5^ However, despite an increase in utilisation, disparities still existed, with inequalities prevalent; thus the NHI prioritised PHC during the first phase of implementation (2012/2013-2016/2017), with the aim of improving the quality of PHC aligned to the National Core Standards of the Office of Health Standards Compliance (OHSC).^2,5^

At all levels of healthcare, including PHC, pharmacotherapy is a frequently used intervention with the appropriate treatment regimen underpinning desired health outcomes.^6^ The knowledge of patient needs, medicine supply and a way of integrating both into safe, effective and appropriate medicine use is required. The pharmacist is ideally placed to support pharmacotherapy through the practice of pharmaceutical care that requires appropriate knowledge and training. Pharmaceutical care is a philosophy based on a patient-centred relationship, with the prioritisation of medicine-related needs and preferences that increase the quality of life of the patient.^6,7^ During the last 40 years, the pharmacy profession has experienced a shift to more cognitive, pharmaceutical care-related roles rather than one focused simply on medicine supply. Importantly, pharmaceutical care complements the principles of PHC, which aim at providing quality, patient-orientated care at the first level of contact of individuals.^8,9,10^

Pharmaceutical care is in contrast to pharmaceutical services as it is a philosophy involving the medicine needs of a patient and provision of the most appropriate medicinal therapy. The dispensing process is a part of pharmaceutical service provision, together with other integral services such as medicine supply management. Dispensing involves three phases including: interpretation and evaluation of a prescription (Phase 1), preparation and labelling of a prescribed medicine (Phase 2) and the provision of information and instructions to the patient (Phase 3). Notably, other than basic counselling on medicine use during Phase 3 of the dispensing process, dispensing offers no tangible patient-specific pharmaceutical care required for positive health outcomes.^8^ Pharmaceutical care involves thorough assessment of patient data, disease data and medicine-related data where patient-specific medicine-related problems are identified, including ineffective dosages, non-adherence, or Adverse Drug Reactions (ADRs). After identifying unmet medicine-related needs, a rational decision-making process follows to develop a prevention strategy for identified problem(s) and establishing a care plan with related interventions. Thereafter, follow-up of established interventions takes place to evaluate the outcome(s) versus the goal(s) of the individualised care plan.^7^

Having a full-time, on-site pharmacist has been presented as the ideal standard of pharmaceutical care provision at PHC facilities, yielding overall cost-saving for health systems.^11,12^ Currently, however, as a result of the shortage of pharmacists in the South African public sector, task-shifting to pharmacist’s assistants and nurses has become common practice in PHC settings.^13^ Task-shifting, a common multidisciplinary phenomenon on the African continent, poses a pragmatic solution to better access and coverage of health service delivery as the demand for PHC increases.^14,15^

Task-shifting of pharmaceutical services has resulted in the emergence of three different dispensing models for the South African PHC. Larger PHC clinics may have an on-site pharmacist supervising pharmaceutical services provided by pharmacist’s assistants, while also being involved in indirectly supervising pharmacist’s assistants or nurses in surrounding clinics. In cases where pharmacists are not available on-site, post-basic pharmacist’s assistants may be responsible for service provision while working under the indirect supervision of a pharmacist (up to five post-basic pharmacist’s assistants supervised by one responsible pharmacist). The dispensing is restricted to quality standards stipulated by Good Pharmacy Practice (GPP). In many PHC clinics where pharmacists or pharmacist’s assistants are not available, pharmaceutical services are provided by nurses who prescribe, dispense and manage medicine supply with the indirect support of a pharmacist or a pharmacist assistant.^10^ Notably however, effects of task-shifting of the dispensing process remains largely unstudied.^16,17^ There is a clear need for research into the impact of task-shifting on efficiency of service provision by contextual influencing factors, such as patient load, understaffing or insufficient infrastructure.^18,19^ Questions have been raised regarding PHC clinics where there is no on-site pharmacist to provide adequate pharmaceutical services and care, leaving dispensing practices misunderstood.^20^ In response to this identified research need, the primary aim of this study was to explore the different dispensing models at three public PHC clinics in the Nelson Mandela Bay Health District. These models included the pharmacist-based dispensing model (a pharmacist on-site with directly supervised pharmacist’s assistants), the pharmacist’s assistant-based dispensing model (with indirectly supervised post-basic pharmacist’s assistants) and the nurse-based dispensing model. The effects of each model on the quality and availability of pharmaceutical services and the perceived quality of pharmaceutical care were studied, with challenges and opportunities relating to the functioning of PHC were identified.

## Method

**FIGURE 1:**
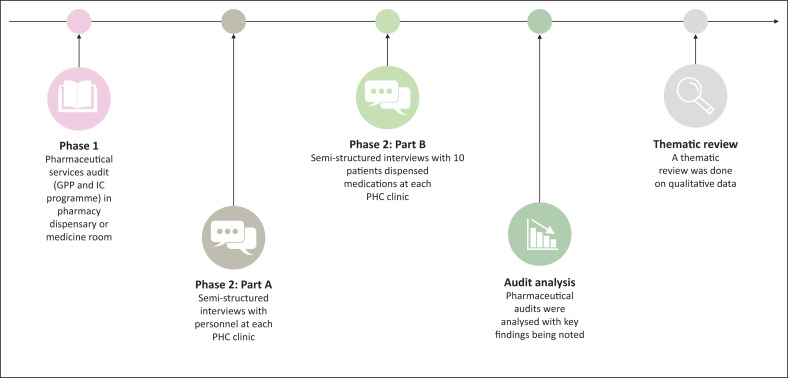
A chronological summary of the study design utilised.

A two-phase, exploratory, mixed-methods study design (as summarised in Figure 1) was utilised to characterise the multifaceted phenomena affecting pharmaceutical services and care provision in each PHC clinic: Clinic N (nurse-based model), Clinic PA (pharmacist’s assistant-based model) and Clinic P (pharmacist-based model).

### Setting

The study took place at three PHC Clinics in the Nelson Mandela Bay District (Sub-District C) in the Eastern Cape Province of South Africa.^21^ Nelson Mandela Bay District is comprised of three sub-districts, namely Nelson Mandela A, Nelson Mandela B and Nelson Mandela C, and falls into socioeconomic quintile 5 with 20.7% medical aid scheme coverage. Within the district there are 39 PHC clinics and 9 community health centres (CHCs) with an estimated 33.3% considered ICs, complying with benchmark IC Core Standards.^22,23^ The Eastern Cape Province is a meaningful setting in which to conduct research related to health service delivery as the healthcare system has a noted history of poor service delivery, in urgent need of critical intervention.^24^

### Study design

#### Phase 1

To provide insight into the quality and availability of pharmaceutical services at each PHC clinic, an audit checklist was compiled. The audit comprised a purpose-designed checklist, integrating aspects of relevant national guidelines; the IC Core Standards; GPP Minimum Standards as stipulated by the Pharmacy Act of 1974; and two checklists provided by the South African Pharmacy Council (SAPC).^10,25,26^ The GPP guidelines form a worthwhile tool in assessing the quality of pharmaceutical service provision at facility level, as wherever these services are provided, the GPP minimum standards should be upheld in practice. As the pharmaceutical services will take place in a PHC clinic context, the IC standards relating to the supply of medicines can also be used to determine the quality of pharmaceutical services provision from a PHC revitalisation reference point. All requirements, based on minimum standards, were checked off as ‘yes/does comply’ or ‘no/does not comply’, with all requirements allocated a numerical weighting (ranging from 1 – ‘not important, but necessary to record’, to 7 – ‘extremely important’) to indicate the importance of each requirement as devised by SAPC checklists.

The audit was compiled with two sections: ‘general standards relating to the premises’ where pharmaceutical services take place and ‘general standards relating to the type of personnel present’, with differing sections relating to the type of personnel dispensing at each facility, i.e. a nurse (in a medicine room), a pharmacist’s assistant (in a pharmacy) and a pharmacist (in a pharmacy). Differing sections were necessary as the GPP standards differ depending on the personnel available to provide pharmaceutical services at a facility. If dispensing takes place by a nurse, there should be a suitable medicine room for medicine storage with dispensing taking place in nurses’ consultation rooms, according to GPP standards. The GPP standards differ for a pharmacy, including whether a pharmacist is on-site or not, as other activities such as compounding or pre-packing of medications may take place in this setting.^10^

The completed audits were analysed by the researcher and totals were added up for each facility. Although no statistical analysis was done on the audit data, the data gave descriptive insight into the general availability and quality of pharmaceutical services offered at each PHC clinic. Additionally, the review of data from Phase 1 was particularly important for Phase 2: Part A and Part B as it provided an opportunity for personnel to elaborate on any audit results that influenced the practice of pharmaceutical services and care at each facility. This speaks to the mixed-methods design of the study with its complementary nature providing synergistic insight relating to key findings.^27,28^

In Phase 1 of the study, all PHC clinics in the Nelson Mandela Bay Health District made up the population of the study. Purposive non-probabilistic sampling was utilised, with three PHC clinics being selected from one sub-district of the Nelson Mandela Bay Health District as made available by the Department of Health. The three PHC clinics were selected on whether they have nurse-based, pharmacist’s assistant-based or pharmacist-based dispensing models. Furthermore, for Phase 1, the inclusion criteria involved the ability to conduct the audits in areas (pharmacy or medicine room) where dispensing, storage and stock management of medicines dispensed at the clinics were carried out.

#### Phase 2

The lived experiences of personnel and patients of pharmaceutical care provision in the dispensing process were investigated in two parts. Firstly, Part A involved semi-structured interviews with the dispensing personnel from each PHC clinic. Thereafter, Part B involved semi-structured interviews with 10 patients who received medication from the personnel at each clinic. This provided valuable insight into the dispensing process from both the dispensing personnel’s and patients’ perception.

For Phase 2: Part A of the study, inclusion criteria included being a pharmacist, nurse or post-basic pharmacist’s assistant involved in the dispensing of medicines at the respective facilities. Exclusion criteria included any staff members who did not dispense medications to patients, as well as nurses facilitating Nurse-Initiated Management of Anti-retroviral Therapy (NIMART) at each PHC Clinic. Nurses dispensing anti-retroviral therapy (ART) certified as part of NIMART have received specialised training to dispense to patients with HIV.^29^ In Phase 2: Part A, the number of participants interviewed at each clinic was restricted by the number of people involved in the dispensing process. For Phase 2: Part B, patients were required to have received dispensed medication at the respective PHC clinic to be eligible for inclusion in the study. Patients who had not had medications dispensed from the clinic were excluded from the sample. Patients who had their medications dispensed through the NIMART process were also excluded.

Each semi-structured interview during Part A and B lasted approximately 10-15 min and took place in a private area. Interviews were conducted in English, with an interpreter available during the interviews to translate questions or responses into IsiXhosa. This was helpful in promoting better insight into patients’ experiences, as some of the participants could provide more articulate responses when speaking IsiXhosa. All interviews were audio-recorded and transcribed for consequent thematic analysis.

### Thematic analysis

After collection and transcription of the recordings in Phase 2: Part A and B, the transcripts were imported and analysed using qualitative data analysis (QDA) software, Atlas.ti®. Firstly, inductive codes were allocated to parts of the transcripts that conveyed a particular analytical meaning. Thereafter, themes were developed from allocated codes that were categorised into groups of related meaning. Conceptual maps or network diagrams were then constructed as a means of graphically linking and relating code groups aiding the thematic analysis and theory building process. The thematic analysis process was independently reviewed for the less biased development of themes.

### Ethical consideration

In the study, the audit and interviews were conducted away from other PHC clinic personnel and patients in a private area to prevent intimidation or harassment by other personnel or staff. Furthermore, all responses were collected anonymously, with the identities of all participants remaining strictly confidential. Participants participated voluntarily in the audits (Phase 1) and the semi-structured-interviews (Phase 2). Before the study commenced, participants were informed of details of the voluntary nature of the research, including pertinent issues of confidentiality and anonymity of information provided. This was done through a printed written consent form, read by the participant and explained by the researcher for purposes of clarity. Once participants had understood and signed this consent form, informed consent of research participants was received before the commencement of interviews.

## Results

### Phase 1

Results from the audit, as displayed in Table 1, provide an indication of the compliance of each clinic with minimum standards of pharmaceutical services.

**TABLE 1:** Results of the audit achieved by each clinic as part of Phase 1 of the study.

Audit checklist section	Achieved score	Maximum score	Percentage
**Clinic N**			
General standards relating to the premises	341	537	-
Standards relating to where the nurse is the member of the personnel (with a medicine room)	54	126	-
Total	395	663	59.5
**Clinic PA**			
General standards relating to the premises	411	537	-
Standards relating to where the pharmacist assistant (post-basic) is the member of the personnel (with a pharmacy)	72	138	-
Total	483	675	71.5
**Clinic P**			
General standards relating to the premises	441	537	-
Standards relating to where the pharmacist is the main member of the personnel (with a pharmacy)	156	210	-
Total	597	747	79.9

The results from Phase 1 indicated that Clinic P was the most compliant (79.9%) with the minimum standards of the audit, with Clinic PA and Clinic N following thereafter with 71.5% and 59.5% respectively. Results identified from the audit relating to crucial aspects of pharmaceutical services provision are mentioned below.

#### Equipment and infrastructure

All facilities had the required equipment to provide necessary services with no immediate concerns regarding medicine storage and medicine integrity. The medicine rooms and dispensaries fulfilled most criteria on the pharmaceutical services audit integral to supporting pharmaceutical services at the facilities.

Although all three clinics provided service to patients partly as per minimum standards, some challenges were noted during Phase 1 that may have compromised the quality of services available. These challenges can be better understood with related responses in Phase 2 as they provide further clarification through lived experiences. These included:

- Poorly designed infrastructure, including small medicine room size and unrestricted access to medicines. For example, the medicine room in Clinic N was small and used as a kitchen for the clinic, meaning that the security of stored medicines was compromised.

The operational manager of Clinic N noted:

‘My biggest challenge is the control or management [*of medicines*]. Let me put it this way, for example, if you can go there now and look at my pharmacy – it’s not a pharmacy, it’s a kitchen at the same time. So I can’t say control of medication is not a challenge.’ (Operational manager [Clinic N])

#### Patient-related challenges

Some challenges noted in Phase 1 limited the effectiveness of pharmaceutical services provided as patient motivation and attitude were often negatively affected:

- Although private consultation rooms were available in all clinics, these rooms are underutilised because of time constraints and a high patient load in Clinics P and PA. Both patients’ and personnel’s responses suggested that dispensing from a dispensing window, even if semi-private, was not always conducive to adequate patient consultation:
  ‘… We try and explain to the person and they will moan that you are taking time. It’s always important to them to come and go quickly. So, if we see that this patient needs it, we call the patient in and explain everything so that you don’t get the shout from the patients behind [*them*].’ (Pharmacist’s assistant 2 [Clinic P])
- With regard to patient record keeping, there was no computerised record keeping or dispensing taking place. Furthermore, pharmacy personnel in Clinic P expressed concern that there was no understanding between clinic staff regarding the system in place to manage patient folders. As a result, patient folders were often lost, incompletely stored or left at home, posing a challenge to providing services based on a complete patient history.

#### Personnel-specific challenges

The personnel dispensing medication reported some challenges that affected their ability to provide pharmaceutical services of the highest quality. These included:

- Concerns over the necessity for dispensing licences or appropriate permits within Clinic N. None of the nurses, except one nurse enrolled in the dispensing course at the time of the study, possessed said licences or permits.
- In relation to the above-mentioned, concerns were raised in Clinic N over the absence of prescription monitoring at the facility, since the diagnosis, prescribing and dispensing of medicines was provided by the same nurse. Thus, medication errors, interactions or health risks might go unnoticed.^30,31^ Where such discrepancies arose in Clinics P and PA, the pharmacist was considered better placed to intervene and, in collaboration with prescribers, to act to rectify the medicine-related problems encountered.
- Nurses and indirectly supervised pharmacy personnel expressed the need for more support from a pharmacist with regard to medicine supply management and dispensing. Although medicine stock-outs were reportedly not common within these three facilities, the need for regular pharmacist support with the management of supply of medicines was considered necessary for constant, reliable access to medicines.

### Phase 2

Pharmaceutical services should ideally be accompanied by appropriate pharmaceutical care provision. Semi-structured interviews were conducted with personnel at each clinic (Phase 2: Part A) (*n* = 11) and with 10 patients who had received medicines from each clinic; (Phase 2: Part B) (*n* = 30) as per inclusion and exclusion criteria.

Table 2 states the types of personnel dispensing medicines who were interviewed as part of Phase 2: Part A of the study.

**TABLE 2:** Number of each type of personnel at each clinic interviewed in Phase 2: Part A of the study.

Clinic	Type of personnel	Number
P	Pharmacist	1
	Pharmacist’s assistant	4
N	Operational manager (registered nurse)	1
PA	Registered nurse	3
	Pharmacist’s assistant	2

**FIGURE 2:**
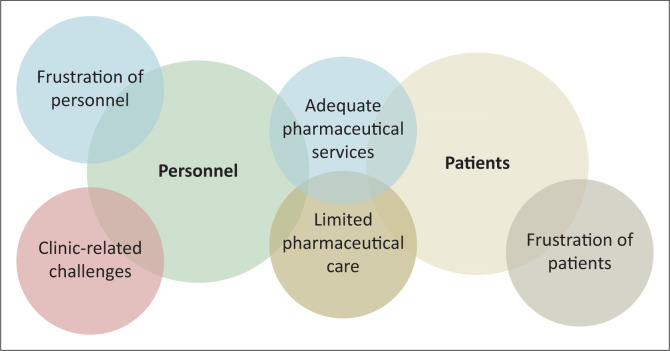
Themes identified from Phase 2: A and B of the study using qualitative data analysis.

The key findings, as per the results of the qualitative data analysis, will be discussed according to the themes identified in Figure 2.

## Clinic-related challenges

- Personnel, particularly in Clinic N, mentioned that patients did not use the PHC clinic system as intended. Patients reportedly bypass their local clinic or use multiple clinics as they prefer some facilities over others, owing to a perception of a difference in the quality of service delivery. This posed a challenge to providing quality services and care as it resulted in fluctuating medicine demand at clinics and difficulty in establishing a relationship with a patient with incomplete patient history or understanding of patient adherence.

## Frustrations of personnel

Personnel noted frustrations that contributed to job dissatisfaction and fatigue, potentially negatively affecting the care provided to patients. Some frustrations noted were:

- Personnel, particularly the nurses (Clinic N), reported a high workload at all facilities, and dispensing services had to be incorporated into an already time-constrained patient consultation. Additionally, indirectly supervised pharmacist’s assistants (Clinic PA) also expressed difficulty in fulfilling dispensing, pre-packing, stock management and prescription monitoring functions simultaneously at the clinic.
- In addition, personnel in all clinics noted staff shortages. Although this shortage may be attributed to a broader human resources deficit within the health sector, it resulted in increased pressure and frustration that may impact on the quality of services provided. For example, a pharmacist assistant stated the following:
  ‘You know what, I like my job. But the problem is we are short staffed, so we are on our own. Like today we are short staffed, so it’s too much for us. We are only two here for the whole clinic.’ (Pharmacist’s assistant 2 [Clinic PA])

## Frustration of patients

- Patients at all clinics were concerned about long waiting times. The personnel in Clinic P were also aware that this is a clear cause of patient frustration that places stress on the pharmaceutical consultation and is a barrier to patient receptiveness:
  ‘You know, we are like the shock absorbers. We are the last team to see the patient. If the patient gets angry starting from security, if they get angry there, with clerks, with nurses, it ends with us. They are already fighting when the situation is like that.’ (Pharmacist’s assistant [Clinic P])

## Adequate pharmaceutical services

Patients at all clinics felt that the pharmaceutical services they received were adequate, fulfilling their medicine supply needs. Although there was satisfaction with the service provision, from both patient and personnel responses in all three clinics, it appeared that Phase 3 of the dispensing process (involving patient-specific medicine-related counselling) was not always provided to patients. Furthermore, many patients expressed the need for more in-depth medicine-related counselling.

## Limited pharmaceutical care

Challenges to providing pharmaceutical care, including patient-centred activities involved in the pharmaceutical care development process, were noted at all three PHC clinics. These challenges included:

- The limited ability of the pharmacist to provide pharmaceutical care owing to a high administrative workload. When asked about his role at the on-site and indirectly supervised clinics, the pharmacist responded:
  ‘My job description is, basically speaking, about 20% dispensing and the rest is stock control and admin[*istration*] work, doing orders and checking orders.’ (Pharmacist [Clinic P])
- At Clinic P, the pharmacist was viewed as an adjunctive provider of medicines and medicine-related information at both the clinic on-site and at indirectly supervised clinics. However, the doctors and nurses do not usually collaborate with the pharmacist as a multidisciplinary healthcare team (MDHT).
- Patient non-adherence was a major theme in all clinics, negatively impacting personnel motivation and the desired treatment outcomes of chronic therapy. When asked about challenges experienced at the dispensary, a pharmacist’s assistant from Clinic P noted:
  ‘The biggest one is that they are defaulting – I can say maybe it’s 80% of patients that are defaulting, but I don’t know why.’ (Pharmacist’s assistant 1. [Clinic P])
- With regard to Adverse Drug Reaction (ADR) monitoring, the pharmacist (Clinic P) in this study had the most knowledge regarding the process. However, he expressed disappointment regarding limited feedback of submitted ADR reports. Other dispensing personnel, in Clinics N and PA, had vague knowledge of ADR reporting, with little reporting happening from within these clinics.

## Discussion

Phase 1 of the study found differing levels of compliance of the medicine room and pharmacies in relation to the minimum standard of pharmaceutical services delivery. In terms of service provision, minimum standards of service were partly upheld in all three PHC clinics; however, the ideal standard was compromised owing to existing challenges. Within the greater context of the NHI and the progressive realisation of access to quality health care, these challenges regarding pharmaceutical services may be of concern.^2^

Firstly, poor infrastructure and its effect on service provision was noted. Within Clinic N, the medicine room was used as a kitchen with unrestricted access. Although dispensing takes place from consultation rooms in Clinic N, the exclusion of a medicine room up to security standards necessary to promote safe storage of medicines means that the mishandling or theft of medicines is a concern. Theft of medicines has been identified as a result of poor medicine supply and security thereof within the South African healthcare system. It promotes potential medicine stock-outs, resulting in medicine unavailability and compromised provision of pharmaceutical services.^32^

Additionally, at Clinics P and PA, the infrastructure supporting patient consultation (in the form of dispensing windows) offered semi-private consultation. There was concern from both patients and personnel as patients felt their privacy was being compromised and that personnel could not properly counsel patients owing to limited time. Although adequate areas for private consultation were available in Clinics P and PA (as required as part of GPP standards), as a result of high patient load, they were often unused.^10^ Andersen, Blenkinsopp and Armstrong^33^ note that privacy involves the ability to have a discussion within the pharmacy setting without being interrupted or overheard by other patients. As part of the dispensing process, providing medicine-related counselling in a private, comfortable setting promotes patient receptiveness and comfort, thus quality provision of pharmaceutical services.^30^ The use of a private consulting area is preferred by patients because of the increased privacy, which is important when dealing with conditions with associated stigma, such as HIV and/or AIDS.^11^ These spaces that allow for patient consultation are particularly important in terms of provision of pharmaceutical care as they affect patient receptiveness within the care relationship. Foster and McIntyre^11^ recommend putting up screens within the waiting areas to ensure privacy for patient counselling while dispensing with less interference from those in the waiting area. This could be a practical solution to preventing consultation interruption.

Some administrative concerns regarding service provision were noted, including inefficient record-keeping practices. In terms of inefficient record keeping, incomplete patient records or missing records were noted as a problem in the clinics. Full patient records are crucial to establishing any previously dispensed medicines and patient history.^10^ Furthermore, patient records can be used to inform knowledge of prescription patterns and common irrational prescribing trends, and can guide demand for medicines, aiding in preventing stock-outs.^34^ Thus, these crucial documents being lost or incomplete at the three clinics was a barrier to providing complete and informed pharmaceutical services. In addition, according to the IC Manual, dispensing processes at PHC clinics should be computerised as part of the District Health Information System (DHIS).^35^ Similarly, the GPP mentions that, if possible, dispensing processes and patient records should be computerised.^10^ At all three clinics there was no computerised system for dispensing, with manual dispensing taking place in each clinic. This observation appears to be in agreement with the situation throughout the country, with Gray et al.^31^ noting that public sector facilities are more likely than private sector facilities to have paper-based dispensing and patient record systems in place.

Lastly, as no dispensing licences or appropriate permits were evident for the nurses involved in the study, concerns over the legal dispensing of medicines was raised. The National Drug Policy of 1996 states that health personnel should be issued with a dispensing licence by the South African Health Products Regulatory Authority (SAHPRA) proving their competency to dispense within particular geographical limits.^36,37^ However, according to the *Medicines and Related Substances Act* (101 of 1965), PHC nurses may be exempt from holding a licence in terms of section 22A (and section 22C[1][a]) if they hold a valid permit in terms of section 56[6] of the *Nursing Act* (Act 33 of 2005).^38,39^ This allows nurses to physically examine and diagnose patients, as well as to keep, supply, administer and prescribe medication for certain conditions in the absence of a doctor or pharmacist.^39^ Gray^37^ notes that this should not be utilised as a long-term resolution and that developing suitable qualifications and regulations and amending the Schedules to the *Medicines and Related Substances Act* to support this provision are necessary when aiming at offering a full set of pharmaceutical services. Thus, better alignment between legislation and practice is required to promote safer and more effective task-shifting processes.

At Clinic N, the diagnosis, prescribing and dispensing of medicines for a patient consultation is provided by the same nurse. Even though permitted by the above provisions in legislation, research has shown that having the prescriber and personnel as the same person at a facility adds to the potential health risk of patients as the ‘double checks’ involved in prescription review (ensuring quality of the process) are bypassed.^11,37^ Thus, medication errors, interactions or health risks may go unnoticed during the provision of pharmaceutical service. Furthermore, the pharmacist’s assistants may face similar challenges in prescription monitoring owing to limited knowledge regarding rational prescribing, medicine interactions or adverse effects.^11,40^ This corresponds to concerns raised in the study as both nurses and pharmacist assistants mentioned the need for more pharmacist support in dispensing activities.

Additionally, in both Clinics N and PA, the need for more support in medicine supply management was expressed. Crowley and Stellenberg^41^ state that because of nurses having an increased workload, they need to be adequately supported such that they can provide adequate pharmaceutical services for patients. Similarly, Osman^40^ notes that indirectly supervised pharmacist’s assistants within PHC settings often find supply management challenging as a result of not having a firm grasp of supply management skills (involving ordering or budget utilisation). Furthermore, many indirectly supervised pharmacist’s assistants, such as those at Clinic PA, are short staffed and lack computerised stock management software to aid with stock management.^40^

From Phase 2 of the study, from both patient and personnel responses, it would appear that Phase 3 of the dispensing process (involving medicine-related counselling) was often inadequate, rushed or left out of dispensing.^10^ However, most patients mentioned they were satisfied with the service as they had received a medicine. It appeared that the act of receiving a medicine at the clinic and the presence of no stock-outs fostered patient satisfaction with provision of pharmaceutical service. However, being provided with an understanding of the relevance and optimal use of the medicine appeared to be comparatively less important. In this regard, it seems patients are not familiar with having consultations with emphasis on their individualised medicine-related needs. Koster et al.^42^ note that patients often believe that they have received complete medicine-related information regarding medicine use; however, some information regarding specific instructions has often been left out of the consultation. Although the three phases of dispensing are defined clearly within the GPP manual, the enforcement of these phases (particularly Phase 3) within every dispensing consultation is not evident.^10^

The strengthening of PHC requires the optimisation of the roles of the current health workforce, in ways that enhance the service availability and quality.^2,5^ However, contextual constraints limited the ability of personnel to practise aspects of this care, such as high patient load and shortages of personnel within the clinics, together with patients not utilising the clinic system as intended. This high workload can hinder the provision of pharmaceutical care as it minimises provider performance and time available for the provision of care during consultation.^43^ High workload is of particular importance because of the consequential effects on clinic staff. Healthcare workers who face high workloads and exhaustion often have poor job satisfaction. Improving the workload of PHC staff (and causative factors, such as staff shortages) is essential to ensuring adequate retention of human resources and job satisfaction.^44,45,46^

Another barrier to the practice of pharmaceutical care was the administrative load of the pharmacist at Clinic P. The pharmacist, the professional best placed to practice pharmaceutical care, had a high administrative load of supporting pharmacist’s assistants at the on-site clinic and at multiple indirectly supervised clinics that limited the amount of time spent on patient-orientated functions.^7,8^ Munroe et al.^12^ suggest that the pharmacist-based model should be the ‘standard of care’ as it has been shown to influence rational prescribing most appropriately, having clinical and cost-saving advantages. Effective task-shifting of this administrative load is necessary to free up the pharmacist for more cognitive, patient-centred activities. In resource-constrained settings, pharmacists are still involved mostly in supply management, as this study confirmed. For example, to facilitate better task-shifting practice, medicine supply management needs to be facilitated by other pharmacy personnel, such that it frees up the pharmacist’s time for pharmaceutical care.^9^ Additionally, the pharmacist in Clinic P was underutilised in MDHT collaboration at PHC level. As a professional with valuable pharmacotherapeutic knowledge, particularly regarding patients with co-morbidities and associated polypharmacy, this underutilisation is a missed opportunity to improve patient care.^1^

Non-adherence, a medicine-related problem, was noted in all three clinics as a concern for dispensing personnel. Non-adherence is of particular concern in the PHC setting owing to the majority of regimens involving long-term chronic therapy.^47,48^ Pharmaceutical care has the ability to improve the adherence of patients through more patient-centred adherence monitoring.^7^ Furthermore, there are also opportunities for pharmacists to play a more decentralised mentorship role. For example, in a study in Lusikisiki within the Eastern Cape of South Africa, an adherence committee was set up to support an ART programme involving community members, clinic committee members, adherence counsellors and clinic staff, meeting monthly.^49^ This concept of an adherence committee including a pharmacist to track adherence and provide medicine-related support could be crucial to solving the problem of high non-adherence. In addition, the pharmacist could support and mentor clinic staff or community health workers (CHWs) on issues relating to adherence of chronic therapy and TB or ART management.^1^

In addition to adherence monitoring, ADR monitoring as part of pharmacovigilance, is a crucial aspect in promoting quality pharmaceutical care. However in the study, the only clinic to have knowledge of this monitoring was Clinic P with an on-site pharmacist.^10^ As with the response of the pharmacist at Clinic P, Suleman^50^ notes that South African health professionals often feel unmotivated to report ADRs appropriately due to poorly disseminated feedback data. This weak knowledge about the importance of ADR reporting in Clinics PA and N may negatively affect health outcomes and the safety of patient populations.^51^ Ideally, pharmacovigilance involves both medicine-related information and clinical knowledge, for example, drawing relationships between a medicine and a reported adverse event. Thus, a decentralised or mentorship role of a pharmacist best placed to combine both medicine-related information and clinical knowledge could see better understanding of medicine safety of differing personnel. This role could provide an opportunity for the pharmacist to advocate for patient safety better within the PHC context, contributing to better quality care and treatment outcomes.^50^

## Limitations

The study took place at three PHC clinics of a sub-district within the Nelson Mandela Bay Health District. Thus, findings may contribute to the understanding of pharmaceutical services and care at the PHC level but cannot necessarily be generalised to other clinics in differing areas or provinces.

## Recommendations

Moving forward requires research, policy and practice changes through the collaboration of researchers, health professionals, policy-makers and patients to promote multidisciplinary and inclusive solutions. Some recommendations proposed from the study include the following.

### Task-shifting optimisation

Task shifting of pharmacy personnel needs to be reconsidered in order to free-up the pharmacist for more patient-centred, cognitive functions. Additional training for pharmacist’s assistants to grow in confidence and ability to execute administrative tasks, such as medicine supply management, should be provided. Optimal skills mix and guidance regarding task-shifting activities should consider the roles and contribution of each health professional in the provision of pharmaceutical services.

### Multidisciplinary team collaboration with PHC clinics

Within the PHC MDHT, there needs to be a better understanding of pharmacists and their input in collaboration regarding patient care. Mechanisms by which the on-site pharmacist may be utilised more regarding pharmacotherapy of patients should be explored further. More emphasis on fostering this sense of multidisciplinary social capital within PHC clinics needs to be explored to maximise the contributions of all members of the healthcare team.

### Revised concept of pharmaceutical care

**FIGURE 3:**
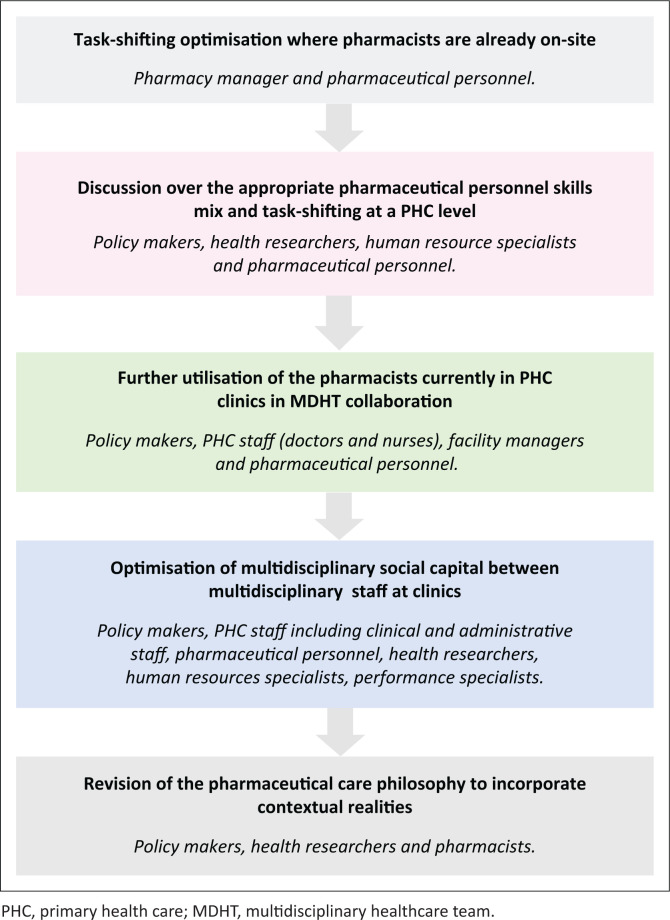
A suggested process of implementation of recommendations and examples of relevant stakeholders involved.

Much has been understood about pharmaceutical care and the need for it in the PHC setting;^11,12^ however, the contextual limitations of facilities limit the ability to provide quality pharmaceutical care. Future policy needs to focus on these particular challenges within resource-constrained settings, providing practical solutions to executing aspects of pharmaceutical care, including a more decentralised, mentorship role of the pharmacist. Figure 3 indicates the suggested process of implementation of the recommendations aimed at utilising the health workforce within current settings, followed by appropriate policy discussion and research contribution to underpin these changes.

## Conclusion

The results of the mixed-methods study indicated that pharmaceutical services partially fulfilled minimum standards with limited pharmaceutical care provision within each dispensing model. The results raised concerns regarding the efforts to improve PHC services under the NHI, as it indicates that there are still many challenges to providing optimal pharmaceutical services within this particular setting. The study also indicated that the current practice of task-shifting compromises the cognitive and collaborative role of the pharmacist, with further opportunities available for the pharmacist to contribute to better patient-centred roles within PHC.
